# Validation of the peroneal nerve test to diagnose critical illness polyneuropathy and myopathy in the intensive care unit: the multicentre Italian CRIMYNE-2 diagnostic accuracy study

**DOI:** 10.12688/f1000research.3933.3

**Published:** 2014-07-21

**Authors:** Nicola Latronico, Giovanni Nattino, Bruno Guarneri, Nazzareno Fagoni, Aldo Amantini, Guido Bertolini, and GiVITI Study Investigators

**Affiliations:** 1 Department of Anesthesia and Critical Care Medicine, University of Brescia at Spedali Civili, Brescia, 25123, Italy; 2 Department of Clinical Epidemiology, IRCCS-Istituto di Ricerche Farmacologiche Mario Negri, Ranica (BG), 24020, Italy; 3 Department of Neuroscience, Section of Clinical Neurophysiology, Spedali Civili, Brescia, 25123, Italy; 4 Department of Anesthesia and Critical Care Medicine, Section of Neuroanesthesia and Neurocritical Care, University of Brescia at Spedali Civili, Brescia, 25123, Italy; 5 Department of Neuroscience, Section of Clinical Neurophysiology, Azienda Ospedaliero-Universitaria Careggi, Firenze, 50134, Italy

## Abstract

**Objectives: **To evaluate the accuracy of the peroneal nerve test (PENT) in the diagnosis of critical illness polyneuropathy (CIP) and myopathy (CIM) in the intensive care unit (ICU). We hypothesised that abnormal reduction of peroneal compound muscle action potential (CMAP) amplitude predicts CIP/CIM diagnosed using a complete nerve conduction study and electromyography (NCS-EMG) as a reference diagnostic standard.

**Design: **prospective observational study.

**Setting: **Nine Italian ICUs.

**Patients: **One-hundred and twenty-one adult (≥18 years) neurologic (106) and non-neurologic (15) critically ill patients with an ICU stay of at least 3 days.

**Interventions:** None.

**Measurements and main results: **Patients underwent PENT and NCS-EMG testing on the same day conducted by two independent clinicians who were blind to the results of the other test. Cases were considered as true negative if both NCS-EMG and PENT measurements were normal. Cases were considered as true positive if the PENT result was abnormal and NCS-EMG showed symmetric abnormal findings, independently from the specific diagnosis by NCS-EMG (CIP, CIM, or combined CIP and CIM). All data were centrally reviewed and diagnoses were evaluated for consistency with predefined electrophysiological diagnostic criteria for CIP/CIM.

During the study period, 342 patients were evaluated, 124 (36.3%) were enrolled and 121 individuals with no protocol violation were studied. Sensitivity and specificity of PENT were 100% (95% CI 96.1-100.0) and 85.2% (95% CI 66.3-95.8). Of 23 patients with normal results, all presented normal values on both tests with no false negative results. Of 97 patients with abnormal results, 93 had abnormal values on both tests (true positive), whereas four with abnormal findings with PENT had only single peroneal nerve neuropathy at complete NCS-EMG (false positive).

**Conclusions: **PENT has 100% sensitivity and high specificity, and can be used as a screening test to diagnose CIP/CIM in the ICU.

## Introduction

Critical illness polyneuropathy (CIP) affects 30% to 50% of the most severely critically ill patients and is the most frequent acute polyneuropathy in the intensive care unit (ICU) ^1^. CIP is often associated with an acute, primary myopathy called critical illness myopathy (CIM), and both conditions occur in patients with multiple organ dysfunctions and failure (MOF). Indeed, CIP and CIM represent the failure of the neuromuscular system in patients with MOF ^1^. CIP classically presents as a sensory-motor axonal polyneuropathy causing difficulty in weaning patients from a ventilator, flaccid limbs, and a possible reduction in deep tendon reflexes. Amplitude reduction of both the compound muscle action potential (CMAP) and the sensory nerve action potential (SNAP) is the predominant electrophysiological finding in CIM and CIP; latency and nerve conduction velocity remain normal or are only slightly decreased. CIM is a primary myopathy with distinctive electrophysiological and morphological findings ^1^. Other features of CIM include increased CMAP duration, normal SNAPs, reduced muscle excitability on direct stimulation and myopathic motor unit potentials on needle electromyography. The clinical features are often the same as for CIP, but sensation, if testable, is normal.

Traditional methods to diagnose CIP and CIM include manual testing of muscle strength using the UK Medical Research Council (MRC) score ^2^ or dynamometry ^3^ to demonstrate severe weakness, electrophysiological tests to explore the function of peripheral nerves and muscles, and muscle biopsy ^1^. Conventional nerve conduction studies (NCS) with measurement of conduction velocity, CMAP and SNAP amplitudes, electromyography (EMG) and other specialised techniques such as direct muscle stimulation or axonal excitability testing may reveal nerve or muscle dysfunction with a high degree of specificity ^4^. However, these techniques require specialised personnel, they are time-consuming and they do not allow diagnosis of small intra-epidermal nerve fiber pathology ^5^. Conventional NCS-EMG may require up to 90 minutes to be completed ^4^. Considering the high prevalence of ICU-acquired neuromuscular disorders, it is unrealistic for conventional NCS-EMG to be used as a large-scale screening tool.

Electrophysiological investigations of peripheral nerves and muscles offer several advantages. They are minimally invasive and easily repeatable, they can be performed at the bedside and the results are immediately available ^6^. Electrophysiological alterations indicating CIP or CIM can also be demonstrated in non-collaborative patients. In comatose patients or in those with persisting sedation or septic encephalopathy who develop severe muscle weakness or paralysis after ICU admission, electrophysiological investigations can be performed to avoid unreasonably pessimistic prognosis by identification of CIP or CIM as the cause ^7^. Electrophysiological alterations are not only essential to establish the diagnosis of CIP and CIM, but they also have an earlier onset than clinical signs or they can be documented at an earlier stage ^7– 11^, thus offering the advantage of a timely diagnosis and the opportunity to conduct potentially valuable interventions before structural muscle-nerve alterations become established ^4^. CMAP reduction is an early event which precedes clinical signs: its onset can be abrupt within 24 hours of normal findings ^10^, and it can be observed as early as 48 hours before clinical signs in patients with sepsis ^12^. Moreover, NCS may also be useful to predict hospital mortality ^9^, and short ^13^ and long-term morbidity ^8, 14, 15^. CMAP after direct muscle stimulation (dmCMAP) may precede the development of ICU-acquired weakness by several days ^11^. Therefore there is a need for a rapid, simple, accurate and valid electrophysiological test to identify CIP and CIM early in ICU.

In a previous study called CRIMYNE ^10^, we found that a simplified electrophysiological investigation, the peroneal nerve electrophysiological test (PENT), had high sensitivity (100%) and moderate specificity (67%) in identifying patients with a diagnosis of CIP or CIM using complete NCS as the reference diagnostic standard. However, the CRIMYNE study had methodological limitations that precluded the acceptance of PENT as a valid screening test in the ICU. Specifically, patients underwent complete NCS only if the PENT was abnormal, thus precluding the possibility of accurately detecting false negative results. Moreover, NCS assessors were not blind to the results of PENT. According to the STARD Statement for reporting studies of diagnostics accuracy, knowledge of the results of the index test can influence the reading of the reference standard, and *vice versa*. Such knowledge is likely to increase the agreement between results of the index test and those of the reference standard, leading to inflated measures of diagnostic accuracy ^16^.

The accuracy of new diagnostic tests should be evaluated before their introduction into clinical practice, because invalid tests may yield exaggerated and biased results, which may trigger their premature adoption in clinical practice and lead to test overuse, increasing patient risks and costs, or to test underuse, missing opportunities to improve health ^17, 18^. Test validation involves comparing the new test with a reference standard, defined as the best available method for establishing the presence or absence of the condition of interest ^17^. Therefore, we designed the multicentre CRIMYNE-2 study according to rigorous methodology to provide an unbiased evaluation of the accuracy of PENT in diagnosing electrophysiologically-proven CIP and CIM diagnosed using complete NCS-EMG as the reference standard.

## Materials and methods

We conducted a prospective observational study between April 2010 and June 2012 in nine medical-surgical Italian ICUs joining the Margherita-Prosafe Project, an international research campaign that collects and analyses clinical data on all patients admitted to the participating ICUs ^19^. The Project, promoted by the GiViTI ( *Gruppo Italiano per la Valutazione degli Interventi in Terapia Intensiva*, Italian Group for the Evaluation of Interventions in Intensive Care Medicine) is based on an electronic Case Report Form (eCRF), which was extended to collect the data of interest for the CRIMYNE-2 study. Two databases were used: Margherita-Due, supported until December 2010, and Prosafe, released on January 2011. Both databases were organized with a core data set and a supplemental module designed for the CRIMYNE-2 research project.

The core data of the Margherita-Prosafe Project included information related to the patients' condition on admission (demographics, admission diagnoses, comorbidities, surgical status), severity-of-illness scoring systems (Simplified Acute Physiology Score [SAPS II], Glasgow Coma Scale [GCS]); procedures, organ failures and complications arising during the ICU; ICU and hospital outcome. The CRIMYNE-2 module included the electrophysiological variables needed to diagnose CIP and CIM, as well as the final diagnosis concluded by the clinical neurophysiologist (see “Possible diagnoses”).

Inclusion criteria were age ≥ 18 years and ICU stay ≥ 3 days, because this patient population is at increased risk of developing CIP and CIM ^1, 20^.

Exclusion criteria were a previous history of neuromuscular disorders or chronic conditions associated with neuromuscular disorders ( *i.e.*, diabetes, renal failure, chronic alcohol abuse), use of neurotoxic or myotoxic drugs, evidence of altered neuromuscular transmission at repetitive stimulation test either caused by neuromuscular blocking agents or disease, lower limb disorders precluding NCS and EMG (for example edema, fractures, amputation, plaster casts), and terminal conditions. Sedation was not an exclusion criterion, and, if present, was not discontinued nor reduced prior to testing.

In each ICU, the inclusion and exclusion criteria were verified daily by the intensivist in charge for the CRIMYNE-2 study. Patients enrolled were subjected to the index test (PENT) and the reference diagnostic standard (NCS-EMG) on the same day by two independent clinicians who were blind to the results of the other test. The research team was allowed to exclude patients if another patient in the same ICU was being concomitantly studied.

## Ethics statement

The study was approved by the Ethics Committee of each participating centre (protocol number 554/2010). Detailed written information was provided to the patients and family members about the study protocol, the scope of research, and the safety of electrophysiological investigations. Written informed consent was obtained from the patient where appropriate. In case of altered consciousness, the Ethics Committees waived the requirement for consent, as in Italy relatives are not regarded as legal representatives of the patient in the absence of a formal designation ^21^. Written informed consent was requested from all surviving patients as soon as they regained their mental competency. All investigations were conducted according to the principles expressed in the Declaration of Helsinki.

### The index test: peroneal nerve test (PENT)

All tests were performed and interpreted by a board-certified technician in clinical neurophysiology with adequate clinical experience.

**Figure 1.**
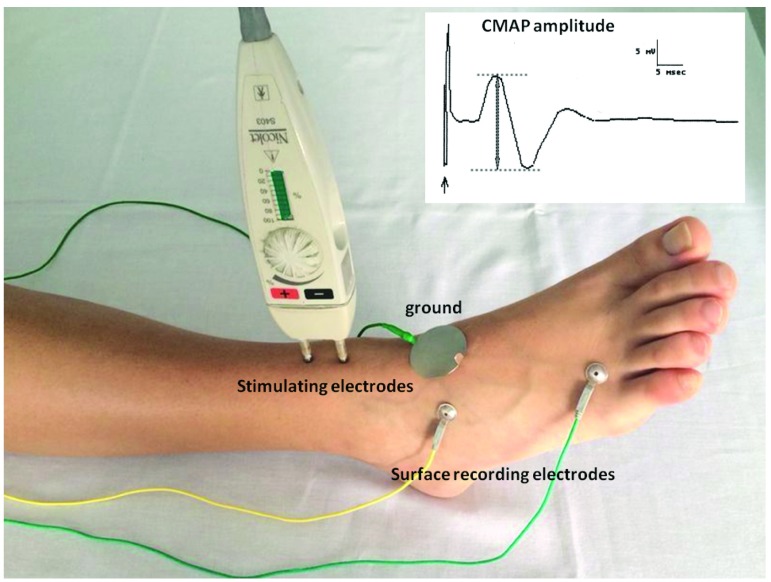
Schematic representation of the peroneal simplified electrophysiological test (PENT). A normal compound muscle action potential (CMAP) amplitude is shown.

PENT started with the measurement of peroneal nerve CMAP amplitude in one leg ( Figure 1). The CMAP was recorded using a pair of surface electrodes: the active electrode was placed on the belly of the extensor digitorum brevis muscle and the indifferent electrode on the distal tendon of the recorded muscle. The peroneal nerve was stimulated over the dorsum of the foot near the ankle, at 7 centimeters from the recording electrodes. The stimulus intensity was gradually increased until the maximal CMAP was obtained. The CMAP amplitude was measured as the maximum voltage difference between the negative and positive peaks ("peak to peak").

If the PENT was normal, the contra-lateral peroneal nerve was investigated. A normal condition was established if the CMAP amplitudes of both peroneal nerves were normal. An abnormal condition was identified if the peroneal nerve CMAP amplitude was reduced below the normal limits of the participating centre in at least one leg.

### The reference diagnostic standard: complete NCS-EMG

NCS-EMG included conduction studies of motor and sensory nerves, and needle EMG, which were performed by board-certified clinical neurophysiologists.

Motor nerves included ^22– 24^: a) the ulnar nerves with recording from the abductor digiti minimi muscle and stimulation at wrist and above the elbow bilaterally; b) the peroneal nerves with recording from the extensor digitorum brevis muscle and stimulation on the dorsum of the foot and below the fibular head bilaterally, and c) the posterior tibial nerves with recording from the abductor hallucis muscle and stimulation at the medial malleolus and popliteal fossa bilaterally. Recorded parameters included the conduction velocity and distal latency, the amplitude of the CMAP and of the recurrent responses after supra-maximal intensity distal stimulation (F response to stimulation of the posterior tibial nerve and of the ulnar nerve).

Sensory nerves included: a) antidromic conduction studies of the ulnar nerves with stimulation at wrist level and recording of 5 ^th^ finger bilaterally, and b) antidromic conduction study of the sural nerves with stimulation at the lower third of the leg and recording at the lateral malleolus bilaterally.

If a neuromuscular transmission defect was suspected, as in case of recent use of neuromuscular blocking agents, low frequency (3Hz) repetitive stimulation studies were performed with stimulation of the right ulnar nerve and recording from abductor digiti minimi and stimulation at wrist. In the case of decremental response on repetitive nerve stimulation, the patient was excluded from the study.

EMG was recorded using a coaxial needle electrode in proximal and distal muscles of upper (brachial biceps, abductor digiti minimi) and lower limbs (quadriceps *f*emori, tibialis anterior). Abnormal spontaneous muscle activity in forms of fibrillation potentials and positive sharp waves was classified in 4 levels (0, 1+, 2+, 3+). Motor units were evaluated in terms of amplitude, duration and morphology and recruitment patterns in patients able to activate their muscles at their own volition ^25, 26^.

Possible diagnoses based on complete NCS-EMG were: CIP, CIM, combined CIP and CIM, undetermined, or normal findings. The diagnostic criteria used conformed to accepted standards and have been described in detail elsewhere ^1, 10, 24^.

We did not evaluate the muscle strength clinically, hence the diagnoses were defined as *probable* ^1^. Probable CIP was established if NCS showed a reduction in the amplitude of CMAP and SNAP below the normal value of the laboratory, with normal or mildly reduced nerve conduction velocity and normal neurotransmission ^1^.

Probable CIM was established in collaborative patients if the CMAP was abnormally reduced below the normal value of the laboratory, the SNAP was normal, and needle EMG demonstrated low-amplitude motor unit potentials with short duration, and early or normal full recruitment, with or without fibrillation potentials ^1^.

In non-collaborative patients, where the differential diagnosis between CIP and CIM could not be established using conventional NCS and EMG ^4, 27^, centres were not required to use specialised neurophysiological techniques such as direct muscle stimulation, and abnormal findings were classified as undetermined.

### Criteria to define true positive and true negative cases

Cases were considered as true negative if both ENG-EMG and PENT assessments were normal. Cases were considered as true positives if the PENT test was abnormal and ENG-EMG showed symmetric abnormal findings, independently from the specific diagnosis at NCS-EMG (CIP, CIM, or combined CIP and CIM). Patients with an abnormal finding at PENT assessment and normal findings or non-symmetrical neuropathy at ENG-EMG ( *i.e.* mononeuropathy or multineuropathy) were considered as false positives.

## Data quality control

All data were centrally reviewed by four of the research team (NL, BG, GN, GB), and diagnoses were evaluated for consistency with predefined electrophysiological diagnostic criteria of CIP and CIM. In the case of discordant results, the centres were contacted and diagnoses were discussed with local personnel until a consensus was reached. If needed, supplemental electrophysiological material was requested from the participating centre and analysed.

### Data presentation, sample size calculation and statistical analysis

The study results are reported according to the Standards for Reporting of Diagnostic Accuracy (STARD) ^17^.

We estimated a prevalence of CIP or CIM of 0.3, a test sensitivity of 90%, and test specificity of 65% based on the results of the CRIMYNE study ^10^. We set a clinically acceptable precision at 10% for estimates of both sensitivity (true positive rate: 85% to 95%) and specificity (true negative rate: 60%–70%), and calculated that 125 patients would be needed to achieve such a precision ^28^.

We described continuous variables as means and standard deviations (SD) or medians and interquartile range (IQR), and categorical variables as counts and percentage.

The 95% confidence intervals (95% CI) for sensitivity and specificity were calculated according to the binomial distribution.

## Results

**Figure 2.**
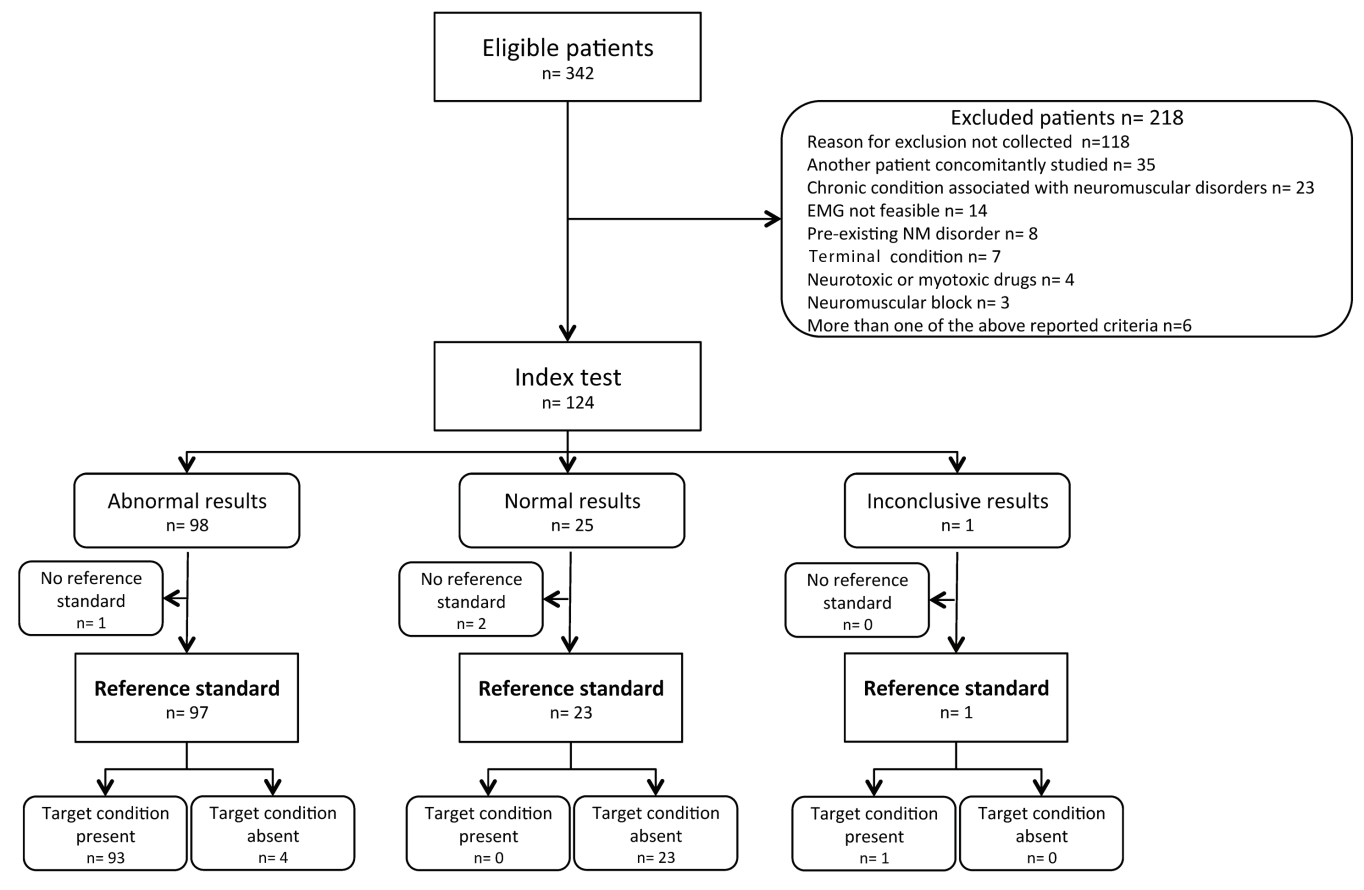
Standards for Reporting of Diagnostic Accuracy (STARD) flowchart.

During the study period, 342 patients were evaluated and 124 (36.3%) were enrolled ( Figure 2).

Discordant results after central revision were documented in five cases. Two cases, classified by the local clinical neurophysiologist as normal despite minimally reduced ulnar CMAP amplitudes, were confirmed as normal at central revision after upgrading the outdated limits of normality used centrally. One case classified locally as abnormal because of minimally reduced proximal peroneal CMAP amplitude (recorded above the fibular head) and normal distal peroneal CMAP (below the fibular head) was classified as normal after central revision, according to the study protocol. One case initially misclassified as having normal PENT because of erroneous transcription of the data was reclassified to abnormal PENT after re-examination of the original electrophysiological data. One case was eventually classified as showing inconclusive results. This patient had normal findings at PENT and abnormal findings at complete NCS-EMG performed 24 hours later. Because this was a protocol violation and CMAP reduction may develop rapidly, within 24 hours of normal findings ^10^, we excluded the patient from the analysis.

Of the 124 patients enrolled, 121 (98%) were investigated with both the index test (PENT) and the reference standard (complete NCS-EMG) with no protocol violation ( Figure 2). The general characteristics of the study population are presented in Table 1. Acute neurologic patients were the main group of patients, with head trauma (35 patients), spontaneous intracranial haemorrhage (25) and aneurysmal subarachnoid haemorrhage (21) being the most frequent causes of admission. Patients had a severe clinical condition, with high prevalence of sepsis, single organ failure (25%) or MOF (62%), and prolonged duration of mechanical ventilation and ICU stay, particularly in patients with abnormal NCS-EMG. Hospital mortality was higher in patients with abnormal NCS-EMG results than in patients with a normal NCS-EMG ( Table 1).

**Table 1.** Demographic characteristics, clinical severity and outcome of the study population (121 patients). GCS: Glasgow Coma Scale; SAPS: Simplified Acute Physiology Score. NCS: nerve conduction study-electromyography.

**Sex** Number (percentage)	Male Female	71 (58.7) 50 (41.3)
**Age (years)** Mean (SD)		51.3 (13.1)
**Surgical status** Number (percentage)	Nonsurgical Emergency surgery Neurosurgery Elective surgery	59 (48.8) 54 (44.6) 38 (70.4) 8 (6.6)
**Trauma** Number (percentage)		37 (30.6)
**Reason for admission** Number (percentage)	Monitoring Intensive treatment	14 (11.6) 107 (88.4)
Organ failures at ICU admission* Number (percentage)	Respiratory failure Neurological failure Cardiovascular failure Renal failure Hepatic failure	106 (87.6) 62 (51.2) 23 (19.0) 11 (9.1) 2 (1.7)
**Cause of ICU admission** Number (percentage)	Neurological Non neurological	106 (87.6) 15 (12.4)
**GCS (first 24 hours)**		7 (5–10)
Median (IQR)		
**SAPS II** Mean (SD)		40.0 (14.0)
**Infections at ICU admission** Number (percentage)	None Present	94 (77.7) 27 (22.3)
**Maximum severity of infection at ICU admission** Number (percentage)	Sepsis Severe sepsis Septic shock	14 (11.6) 6 (5.0) 7 (5.8)
**Infections during the ICU stay** Number (percentage)	None Present	50 (41.3) 71 (58.7)
**Maximum severity of infection during ICU stay** Number (percentage)	Sepsis Severe sepsis Septic shock	30 (24.8) 37 (30.6) 18 (14.9)
ICU procedures*	Mechanical ventilation Vasoactive drugs Tracheostomy Enteral nutrition Parenteral nutrition	115 (95.0) 78 (64.5) 72 (59.5) 106 (87.6) 47 (38.8)
**ICU stay (days)** Median (IQR)	All patients Patients with abnormal NCS Patients with normal NCS	19 (13–33) 23 (16–39) 13 (10–19)
**Hospital stay (days)** Median (IQR)	All patients Patients with abnormal NCS Patients with normal NCS	32 (21–43) 33 (24–46) 22 (15–30)
**Hospital mortality** Number (percentage)	All patients Patients with abnormal NCS Patients with normal NCS	22 (18.2) 20 (21.3) 2 (7.4)

* Percentages may sum to more than 100% because patients may be classified in more than one category.

Of 23 patients with normal NCS-EMG, all also had normal findings with PENT with no false negative results ( Figure 2). Of 97 patients with abnormal ENG-EMG, four had peroneal nerve mononeuropathy at complete NCS-EMG and abnormal findings at PENT, and were classified as false positives. The sensitivity of the PENT was 100% (95% CI 96.1-100.0) and the specificity 85.2% (95% CI 66.3-95.8). Sensitivity was comparable to that found in the CRIMYNE study (100%). Specificity was higher in this study compared to the CRIMYNE study, but the difference was not statistically significant (CRIMYNE: 67%, CRIMYNE-2: 85%, p=0.08).

The electrophysiological diagnosis in the 93 patients with abnormal findings at complete NCS-EMG was CIP in 35 patients (37.6%), combined CIP and CIM in 16 (17.2%) patients and undetermined in 42 (45.2%) patients.

Electrophysiological investigations were performed on median ICU day 9 (IQR 5-16). The median time needed to perform PENT and the complete NCS-EMG was 10 minutes (IQR 8.0-10.5) and 50 minutes (40–60). In 85 cases (70.2%), the peroneal nerve examined first was abnormal, thus eliminating the need for contralateral testing.

Data set for the CRIMYNE-2 study on the validation of perineal nerve test to diagnose polyneuropathy and myopathy in 121 patients The data show the characteristics and the outcomes of the patients enrolled in the CRIMYNE-2 study. surgicalStatus: surgical status on admission (nonSurgical= non surgical, emergSurgical= emergency surgical, electSurgical= elective surgical). surgicalId_neurosurg: patient undergoing neurosurgery in the 7 days preceding or in the 24 hours following ICU admission (1=yes, 0=no). Trauma: admission for a recent trauma (<1 week). admReas: reason for admission (monitWean= monitoring/weaning, intTreat= intensive treatment). neurologicalPatientAdm: patient admitted with a neurological condition on admission (1= yes, 0= no). gcsAdm: Glasgow Coma Scale on admission. sapsIIadm: SAPSII on admission. sevInfections: severity of infection on admission (0= not infected on admission, 1=infection with or without SIRS, 2=SEVERE SEPSIS, 3=SEPTIC SHOCK). maxSevInfections: maximum severity of infection during the stay (0= never infected during the stay, 1=infection with or without SIRS, 2=SEVERE SEPSIS, 3=SEPTIC SHOCK). InconclusiveResult: whether the result were inconclusive or not (see STARD flowchart, 1= yes, 0= no). PENT: result of the peroneal nerve electrophysiological test. NCS: result of the complete nerve conduction study. EMG: result of the electromyography.

## Discussion

In this multicentre diagnostic accuracy study, we found that the PENT had 100% sensitivity and high specificity in diagnosing CIP or CIM, confirming the preliminary results of the previous CRIMYNE study ^10^. The time needed to complete the test was 10 minutes, which was considerably shorter than the time needed to complete NCS-EMG (50 minutes).

Conventional NCS and EMG have never gained popularity in the ICU because they are time-consuming, not readily available, expensive, and technically challenging as they require specialised personnel. Therefore, our results may be important in promoting a wider use of electrophysiological investigations in the ICU. As a validated, high-sensitivity, minimally invasive, non-volitional and quick diagnostic test, PENT can accurately exclude CIP or CIM if the result is normal, and can be proposed for the assessment and monitoring of the neuromuscular function in the early stages of critical illnesses.

Abnormal PENT cannot discriminate between CIP, CIM or combined CIP and CIM; in fact, in our study, diagnosis was undetermined in almost half of patients. Because precise definition of pathology can be relevant for predicting the recovery of muscle strength ^13, 14^, PENT can be used as a screening tool to select patients deserving further investigations. Moreover, “false positive patients” are those with peroneal nerve mononeuropathy who are worthy of medical attention. Involvement of the peroneal nerve is invariably detected in patients with CIP or CIM, possibly because it is the longest nerve of the body and, hence, it is mostly vulnerable to the energy deficit caused by tissue ischemia or dysoxia ^7, 10^. In fact, the biosynthesis takes place in the neuronal cell body and then the structural components are moved into the axons and transported to their final destination to generate the action potential and to maintain axonal integrity, a process that requires considerable amount of energy ^7, 10^. This might explain the reason why reduced peroneal nerve CMAP is highly sensitive compared to other tests. Pragmatically, measurements of peroneal CMAP could be implemented to diagnose all patients with ICU-acquired neuromuscular disorders, either CIP or CIM, while other tests, such as measurement of dmCMAP, could be used to differentiate CIM from CIP ^29^. Recent studies suggest that the *duration* of the CMAP can be prolonged in patients with CIM ^30^.

Five single-centre studies have used simplified electrophysiological tests of neuromuscular function in the ICU ^10, 11, 31– 33^. In two randomized controlled trials on intensive insulin treatment ^31, 32^, the diagnosis of CIP was suggested by the presence of abundant spontaneous activity in the form of positive sharp waves and fibrillation potentials on EMG ^34^. No reference diagnostic standard was used, thus the sensitivity and specificity of the test are unknown. In the CRIMYNE study ^10^, we used the same index test and reference diagnostic standard of the CRIMYNE-2 study, but comparison was unblinded and was limited to patients with abnormal findings at the index test, which may have inflated the test sensitivity. In a prospective observational study in surgical ICU patients ^11^, the sensitivity and specificity of dmCMAP in predicting the development of ICU-acquired weakness were 83.3% and 88.8%, respectively. In a more recent study ^33^, sensitivity and specificity of combined peroneal nerve CMAP and sural nerve SNAP evaluations were 100% and 81%, respectively. However, in both studies all electrophysiological measurements were performed by the same investigators, and no blind assessment of the reference diagnostic test was implemented ^35^.

Among non-volitional non-electrophysiological tests, ultrasound-guided assessment of muscle mass can reliably detect early muscle changes. In a recent prospective cohort study in 63 critically ill patients who were developing acute muscle wasting, the rectus femoris cross-sectional area decreased significantly from days 1 to 7 by 12.5% ^36^. Simultaneous measurement of muscle strength and muscle mass has been suggested as a critical step in all future studies investigating ICU-acquired weakness ^37^. However, assessing muscle strength may be difficult in the early stage of acute disease, and the use of PENT could be of value in assessing the generation of the action potential, which is an essential pre-requisite for muscle contraction.

The prevalence of electrophysiological abnormalities was 76%, which is higher than reported in a systematic review [46% (95% C.I. 43–49%)] ^20^. We enrolled patients with high incidence of sepsis and MOF, and prolonged mechanical ventilation ^5^. It is therefore not surprising that this patient group also had a high incidence of ICU-acquired neuromuscular disorders that are currently viewed not as isolated events, but rather as an integral part of the process leading to MOF ^1^. In two recent studies, severe muscle weakness was documented in 74% of patients ^38^ and electrophysiological abnormalities in 87% ^11^. In an unselected population of critically ill children, the occurrence of generalised muscle weakness was substantially lower (1.7%) ^39^, reflecting the need for future studies to include much larger samples of unselected ICU patients to evaluate the true incidence of ICU-acquired neuromuscular disorders.

Several limitations of the current study are worth discussing. First, we did not test muscle strength clinically, and therefore, we could not evaluate the proportion of patients with pure electrophysiological alterations that developed definite CIP or CIM. Current recommendations suggest that patients with a MRC sum-score of less than 48 or reduced handgrip dynamometry should undergo physical rehabilitation without any further testing ^1, 40^. NCS and EMG testing should be reserved for those patients not improving despite receiving such treatment. MRC and dynamometry can be easily implemented in the ICU, and inter-observer reliability is good. However, volitional tests require the patient’s collaboration, hence an inability to perform the test or the recording of low values may occur as a result of coma, delirium, sedation, injury, or simply poor patient motivation or attention. Recent studies suggest that manual muscle testing can be insufficient for the early detection of ICU-acquired neuromuscular disorders in most patients ^41^. In fact, ascertainment of ICU-acquired weakness at ICU day 7 post-awakening, as originally defined ^2^, can be of limited value because many patients have been already discharged from the ICU at that time, reflecting the change in clinical ICU practice towards earlier discharge ^38^. Moreover, inter-observer agreement on MRC sum-score assessment is only moderate in patients with MRC scores that are lower than 48, probably because the ability to perform the volitional MRC sum-score among the most severe patients is variable ^38, 42^. PENT is not dependent upon patients’ collaboration and could be performed at an early stage to detect initial functional derangement and to use the results to start investigational treatments with the aim of interrupting pathological mechanisms at their onset ^43^. Several studies have demonstrated that electrophysiological alterations are followed by muscle weakness, but they included a small number of selected ICU patients ^7– 11^. PENT is a quick test and could be used in large ICU populations.

Second, the large majority of patients were critically ill neurologic patients, which may limit the generalisability of the results to other ICU populations. However, development of CIP and CIM is independent from the cause of ICU admission, and it rather depends on MOF, which is also a common complication in acute neurologic patients ^44^, as it is confirmed in this study.

Lastly, we excluded patients with diabetes, who may represent up to 16% percent of patients admitted to the ICU ^45^. Peripheral neuropathy is a common complication in diabetic patients ^46^. However, generalised symmetric polyneuropathy typically involves sensory nerves with mild reduction in distal sensory response amplitudes (e.g., those of the sural nerves) ^47^. In contrast, motor response amplitudes, as those tested with peroneal nerve evaluation, are generally preserved and decrease only in more advanced disease.

## Conclusions

To the authors’ knowledge, this is the first time that a simplified electrophysiological test to assess the peripheral nerve and muscle function has been evaluated in an ICU population using a rigorous methodology based on independent and blinded comparison with a reference standard.

Measurement of CMAP of the peroneal nerve showed 100% sensitivity and high specificity in diagnosing probable CIP or CIM, and did not require patients’ collaboration. Potential useful applications of the test can be at the early ICU stage, when volitional tests can be rarely performed. At a later stage, before discharge from ICU or acute-care hospital, a normal test excludes CIP or CIM and the need for further electrophysiological investigations. If patients present abnormal values, they might have CIP, CIM or focal peripheral nerve complications such as peroneal entrapment neuropathy that warrant neurological consultation.

The application of this test to wider populations of ICU patients might allow a more precise estimation of the true incidence of ICU-acquired neuromuscular disorders. With adequately powered observational cohort studies, future studies might be able to evaluate the relationship between electrophysiologically-proven CIP or CIM and reduced muscle strength and mass in the ICU ^36, 37^, as well the relationship between these acute changes and persistent muscle weakness or persistent physical dysfunction at long-term follow-up ^42^.

## Data availability

*figshare*: Data set for the CRIMYNE-2 study on the validation of peroneal nerve test to diagnose polyneuropathy and myopathy in 121 patients, doi: http://dx.doi.org/10.6084/m9.figshare.1021506 ^48^

## Consent

Detailed written information was provided to the patients and family members about the study protocol, the scope of research, and the safety of electrophysiological investigations. Written informed consent was obtained from the patient where appropriate. In case of altered consciousness, the Ethics Committees waived the requirement for consent, as in Italy relatives are not regarded as legal representatives of the patient in the absence of a formal designation ^21^. Written informed consent was requested from all surviving patients as soon as they regained their mental competency.
