# Supplementary material for: Validation of the peroneal nerve test to diagnose critical illness polyneuropathy and myopathy in the intensive care unit: the multicentre Italian CRIMYNE-2 diagnostic accuracy study
Source: F1000Res. 2014 Jul 21;3:127. Originally published 2014 Jun 11. [Version 3] doi: 10.12688/f1000research.3933.3 (PMC4184363; doi:10.12688/f1000research.3933.3)
Supplement: Peer review history

# Peer review: Referee response for version 3

**DOI:** 10.5256/f1000research.5168.r5088

**Published:** 2014-08-22

**Authors:** Werner J. Z'Graggen

**Affiliations:** 1 Department of Neurology, Inselspital, Bern University Hospital and University of Bern, Bern, Switzerland

The authors have addressed my queries.

I have read this submission. I believe that I have an appropriate level of expertise to confirm that it is of an acceptable scientific standard.

# Peer review: Referee response for version 3

**DOI:** 10.5256/f1000research.5168.r5560

**Published:** 2014-07-23

**Authors:** Alexander Semmler

**Affiliations:** 1 Department of Neurology, University Hospital Zurich, Zurich, Switzerland

I have read this submission. I believe that I have an appropriate level of expertise to confirm that it is of an acceptable scientific standard.

# Peer review: Referee response for version 2

**DOI:** 10.5256/f1000research.4851.r5212

**Published:** 2014-06-23

**Authors:** Alexander Semmler

**Affiliations:** 1 Department of Neurology, University Hospital Zurich, Zurich, Switzerland

Title, abstract, study design, methods are adequate. Results are sound.

Minor comments:

1. The authors compared PENT with standard nerve conduction studies. The diagnostic criteria for CIM/CIP include clinical testing and muscle biopsy. Please discuss why you have not compared your method to the diagnostic standard.
2. The typical patient populations on ICUs are different from the population of the actual study. Patients are often old, and show a high prevalence of diabetes, alcohol abuse and other medical conditions that predispose to polyneuropathy. This may lead to a lot more false positives when only PENT is performed. Please discuss that.
3. Please speculate how PENT could influence the current standard medical practice. 

I have read this submission. I believe that I have an appropriate level of expertise to confirm that it is of an acceptable scientific standard.

# Response

**Published:** 2014-07-21

**Authors:** Nicola Latronico

**Affiliations:** University Department of Anesthesia and Critical Care Medicine, University of Brescia, Italy

1. *The authors compared PENT with standard nerve conduction studies. The diagnostic criteria for CIM/CIP include clinical testing and muscle biopsy. Please discuss why you have not compared your method to the diagnostic standard.*
  The CRIMYNE-2 study was a validation study comparing electrophysiological tests, and hence we did not evaluate the muscle strength clinically nor muscle histology, as we declared in the Methods. Possible diagnoses with PENT were normal or abnormal condition with no inference to the differential diagnosis between CIP and CIM. Hence, PENT can only be used as a screening tool, and we further specified this aspect in the Abstract’s conclusion as suggested by the other reviewer. As a high sensitivity test, PENT has a high negative predictive value and is particularly useful in excluding diseased patients if normal. If abnormal, further neurological, electrophysiological and biopsy investigations can be needed to exactly define the nature of the disease.
2. *The typical patient populations on ICUs are different from the population of the actual study. Patients are often old, and show a high prevalence of diabetes, alcohol abuse and other medical conditions that predispose to polyneuropathy. This may lead to a lot more false positives when only PENT is performed. Please discuss that.*
  This is an important topic, and we thank to reviewer for giving us the possibility to further discuss it. As we mentioned in the Discussion, the diabetic neuropathy typically involves sensory nerves, motor nerves are involved only in the advanced stage of the disease. Thus, diabetic patients with abnormal PENT would deserve further evaluation. In patients with abnormal PENT, clinical history together with neurological and electrophysiological investigations and appropriate follow-up are essential to identify those patients in whom the CIP complicates a pre-existing diabetic neuropathy. It is worth noting that most of these patients discharged from the ICU are currently left with undefined diagnosis.
3. *Please speculate how PENT could influence the current standard medical practice.*
  As a quick, noninvasive and easily repeatable test, PENT can be used as a screening test in all ICU patients with severe critical illness, such as those with protracted ICU stay or mechanical ventilation, those with sepsis and multiple organ dysfunctions or those with clinically identifiable ICU-acquired weakness. This would be a major change in current practice, because, as mentioned above, many critically ill patients do not receive adequate evaluation of acute neuromuscular problems arising during the ICU possibly as a consequence of rapid turnover or other causes [ref-1]. As a screening test, PENT may select patients with normal finding who do not require further investigations. Moreover, patients with ICU-acquired weakness have a better outcome if electrophysiology is normal [ref-2]. Patients with abnormal PENT will not invariably have CIP or CIM. With further evaluation, they may eventually be diagnosed with peroneal nerve palsy or other peripheral nervous system diseases that merit medical attention. In prospect, electrophysiological alterations of peripheral nerves and muscles may help in instituting timely treatments since they may precede muscle weakness and functional impairment or their diagnosis can be established at an earlier stage [ref-3]. Patients in the ICU often are sedated or develop delirium and consciousness disturbance or have acute neurological illnesses precluding the use of clinical tests such as the Medical Research Council score or handgrip dynamometry early in the ICU. 

[References]

[[1|type=journal|title=Clinical predictive value of manual muscle strength testing during critical illness: an observational cohort study. |authors=Connolly/BA;Jones/GD;Curtis/AA;Murphy/PB;Douiri/A;Hopkinson/NS;Polkey/MI;Moxham/J;Hart/N|source=Crit Care.|year=2013|vol=17|issue=5|fpage=R229 |pmid=24112540|pmcid=PMC4057053 |doi=10.1186/cc13052]]

[[2|type=journal|title= Screening for critical illness polyneuromyopathy with single nerve conduction studies|authors=Moss/M;Yang/M;Macht/M;Sottile/P;Gray/L;McNulty/M;Quan/D|source=Intensive Care Med.|year=2014|vol=40|issue=5|fpage=683 |lpage=690|pmid=24623137|doi=10.1007/s00134-014-3251-6]]

[[3|type=journal|title=Use of electrophysiologic testing|authors=Latronico/N;Shehu/I;Guarneri/B |source=Crit Care Med.|year=2009|vol=37|issue=10|fpage=S316|lpage=S320|pmid=20046116|doi=10.1097/CCM.0b013e3181b6f9f3]]

# Peer review: Referee response for version 2

**DOI:** 10.5256/f1000research.4851.r5182

**Published:** 2014-06-19

**Authors:** Werner J. Z'Graggen

**Affiliations:** 1 Department of Neurology, Inselspital, Bern University Hospital and University of Bern, Bern, Switzerland

Latronico and colleagues report the findings of the CRIMYNE-2 study. The preceding CRIMYNE study showed that a simplified electrophysiological investigation (limited to a peroneal nerve conduction study (PENT)) has a sensitivity of 100% and specificity of 67% for the detection of a critical illness polyneuropathy (CIP) and/or critical illness myopathy (CIM) compared to a state of the art electrophysiological investigation. The CRIMYNE-2 study aimed to validate these findings, since the preceding study had some important methodological limitations, which was probably one of the reasons why this simplified approach was not widely accepted by clinicians and researchers. To my opinion, the CRIMYNE-2 study is well designed and achieves its aim. The study was carefully done and confirmed for PENT a sensitivity of 100% and a specificity of 85%. There were no false negative results. The authors conclude that PENT can be used as a screening tool for CIP and CIM and that PENT might be more reliable than other screening methods currently performed, since no patient collaboration is needed.

There are some study limitations that should be discussed by the authors in the paragraph about limitations (to some extent the authors mention them in other parts of the manuscript). Based on the current diagnostic criteria for CIM, state of the art electrophysiological investigations allow only the diagnosis of probable CIM, definite diagnosis needs additionally the presence of weakness and histological changes. For CIP, definite diagnosis requires in addition clinical weakness. CRIMYNE-2 did not compare PENT with the criteria for definite CIP/CIM, which could have caused an additional study bias. Furthermore, the authors propose to use PENT for screening without additional repetitive stimulation (which they used in the study). This could lead to an increase of false positive results. Also this aspect should be discussed.

The results of CRIMYNE-2 demonstrate that PENT can be used as a screening tool for CIP/M. PENT cannot be used to discriminate between CIP and CIM or concomitant CIP and CIM. The authors should therefore state in their conclusion in the abstract, that PENT can be used as screening test.

I have read this submission. I believe that I have an appropriate level of expertise to confirm that it is of an acceptable scientific standard.

# Response

**Published:** 2014-07-21

**Authors:** Nicola Latronico

**Affiliations:** University Department of Anesthesia and Critical Care Medicine, University of Brescia, Italy

We thank the reviewer for his thoughtful comments. As mentioned in our reply to Dr. Semmler, PENT was conceived as a screening tool, and diagnoses were categorized as normal or abnormal condition with no inference to the differential diagnosis between CIP and CIM. We modified the conclusions in the Abstract to clarify even further this concept, as required by the reviewer.
